# Bilateral Sciatic Nerve Compression Due to Abnormal Termination of the Small Saphenous Vein Into the Popliteal Vein: A Rare Mechanism of Pseudoclaudication of the Lower Extremities

**DOI:** 10.7759/cureus.10543

**Published:** 2020-09-19

**Authors:** Byron Chalidis, Dimitrios Kitridis, Maria Tirta, Panagiotis Givissis

**Affiliations:** 1 School of Medicine-1st Orthopaedic Department, Aristotle University of Thessaloniki, Thessaloniki, GRC; 2 School of Medicine, Aristotle University of Thessaloniki, Thessaloniki, GRC

**Keywords:** sciatic nerve, claudication, popliteal fossa, short saphenous nerve, popliteal vein, entrapment

## Abstract

Claudication in the young population is a rare condition derived from varied neurogenic and vascular conditions. We report a case of bilateral claudication of the lower extremities in a young athlete after intense training. Apart from tenderness to deep palpation of the popliteal fossa, physical and laboratory examinations did not reveal any abnormal findings. According to the patient’s symptoms, sciatic nerve entrapment to the popliteal fossa was suspected and bilateral surgical exploration of the sciatic nerve was performed. The sciatic nerve was found to be entrapped before its division to the tibial and common peroneal nerves from the terminal branch of the small saphenous vein (SSV) into the popliteal vein (PV). The terminal section of SSV was subsequently ligated and resected to relieve the pressure on the sciatic nerve. One year after surgery, the patient was able to run long distances as well as sprint and train without any restriction. Rare conditions may lead to pseudoclaudication in young individuals and athletes during exercise. Normal physical and laboratory tests must raise the suspicion of sciatic nerve compression from fibrous bands or abnormal anastomotic veins that may also exist bilaterally. Surgical exploration along with sciatic nerve release remains the only treatment solution when conservative treatment fails to alleviate the symptoms.

## Introduction

Claudication is a quite uncommon condition in the young patient population that frequently is developed during exercise and is relieved by a short period of rest. It may result from various conditions, such as medial tibial stress syndrome, stress fracture, chronic exertional compartment syndrome, popliteal artery entrapment syndrome, and nerve entrapment [1]. The latter pathology includes mainly the common peroneal nerve, the superficial peroneal nerve, and the saphenous nerve [2]. Sciatic nerve entrapment is even rarer and may occur from the pelvis to the distal thigh region and more often between the greater sciatic notch and the ischial tuberosity [3]. Nerve compression is usually a result of scar formation following surgery, trauma, or infection but other uncommon conditions such as bone or soft tissue masses (i.e., bony impingement, tumor, abscess, hematoma), fibrous bands and vascular leashes may also lead to claudication [3]. 

We report a rare case of pseudoclaudication of the lower extremities due to bilateral sciatic nerve entrapment from the termination of the small saphenous vein (SSV) into the popliteal vein (PV). According to our knowledge and literature review, no similar cases have been previously reported.

## Case presentation

A 20-years old male sprinter presented to our department complaining of sudden pain in both lower limbs along with painful calf cramps and feet numbness after intense training. Symptoms were apparent for more than three months even after running less than 100 meters and it severely affected his overall sports performance.

Physical examination revealed no motor deficit in both legs. Skin and temperature sensation determined with a swab or pinching and an alcohol-soaked gauze, respectively, were normal. Peripheral pulses were palpable in the posterior tibial and dorsalis pedis arteries; the ankle-branchial index (ABI) was 1.0 in both legs (normal values: 0.9-1.2). The above parameters remain unchanged after the treadmill walking test. However, while running on the treadmill, the skin sensation below the knees was reduced. The pinch test of plantar and dorsal feet was also abnormal. Deep palpation of the popliteal fossa was extremely painful and the pain radiated toward the feet but the Tinel’s sign in popliteal fossa was negative. The ABI values remained stable in both legs. Magnetic resonance imaging (MRI) of both knees did not reveal any abnormal findings. Electromyography (EMG) test showed normal function of all nerves of the lower extremities. Similarly, digital subtraction angiography (DSA) revealed normal popliteal artery flow bilaterally. According to the symptoms and the laboratory findings, sciatic nerve entrapment to the popliteal fossa was suspected and after a thorough discussion with the patient, consent was obtained for surgical exploration of the sciatic nerve.

**Figure 1:**
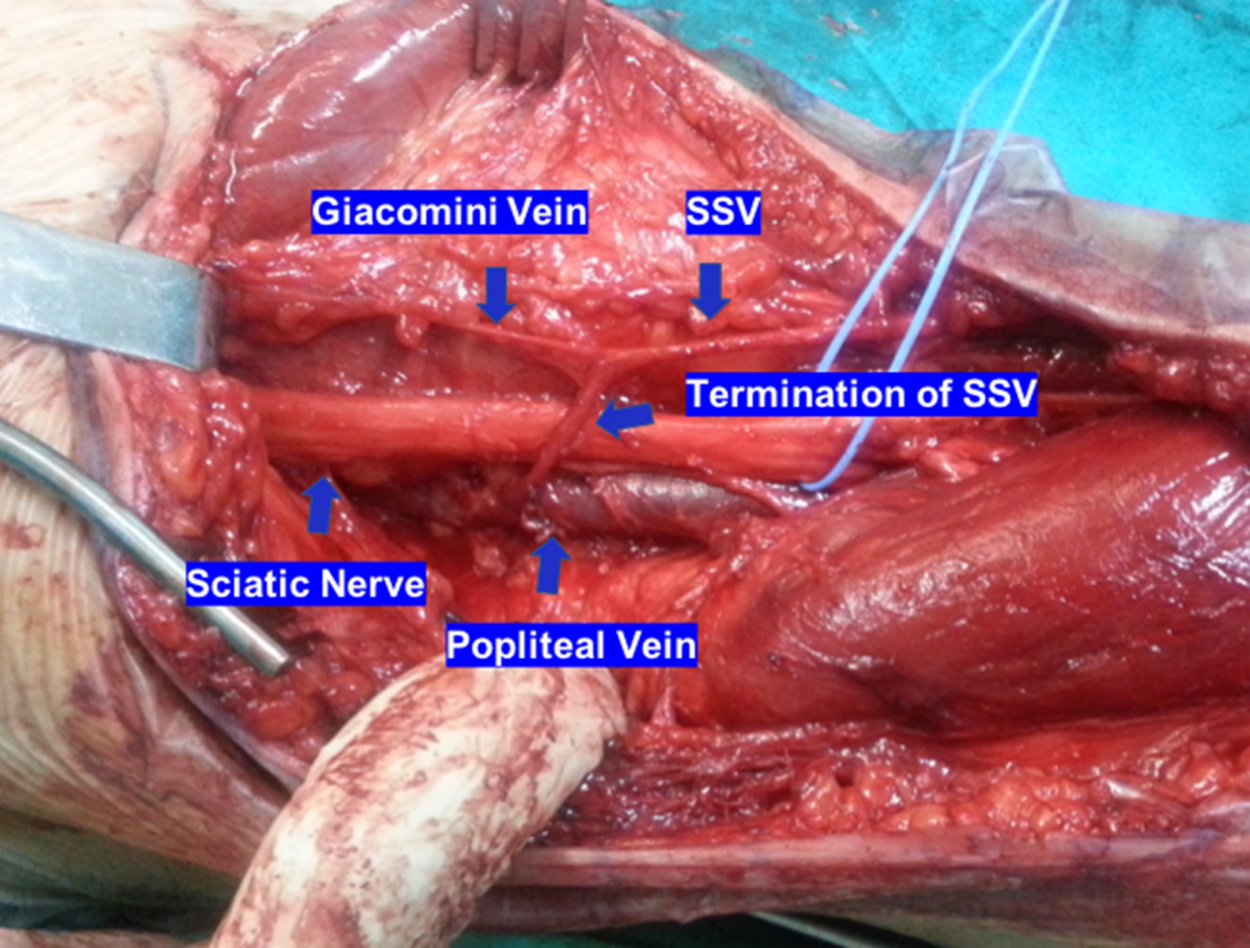
Sciatic nerve compression in the sapheno-popliteal junction. The termination of the small saphenous vein (SSV) runs obliquely and above the sciatic nerve before it drains into the popliteal vein. Part of the SSV continues proximally as the Giacomini vein

**Figure 2:**
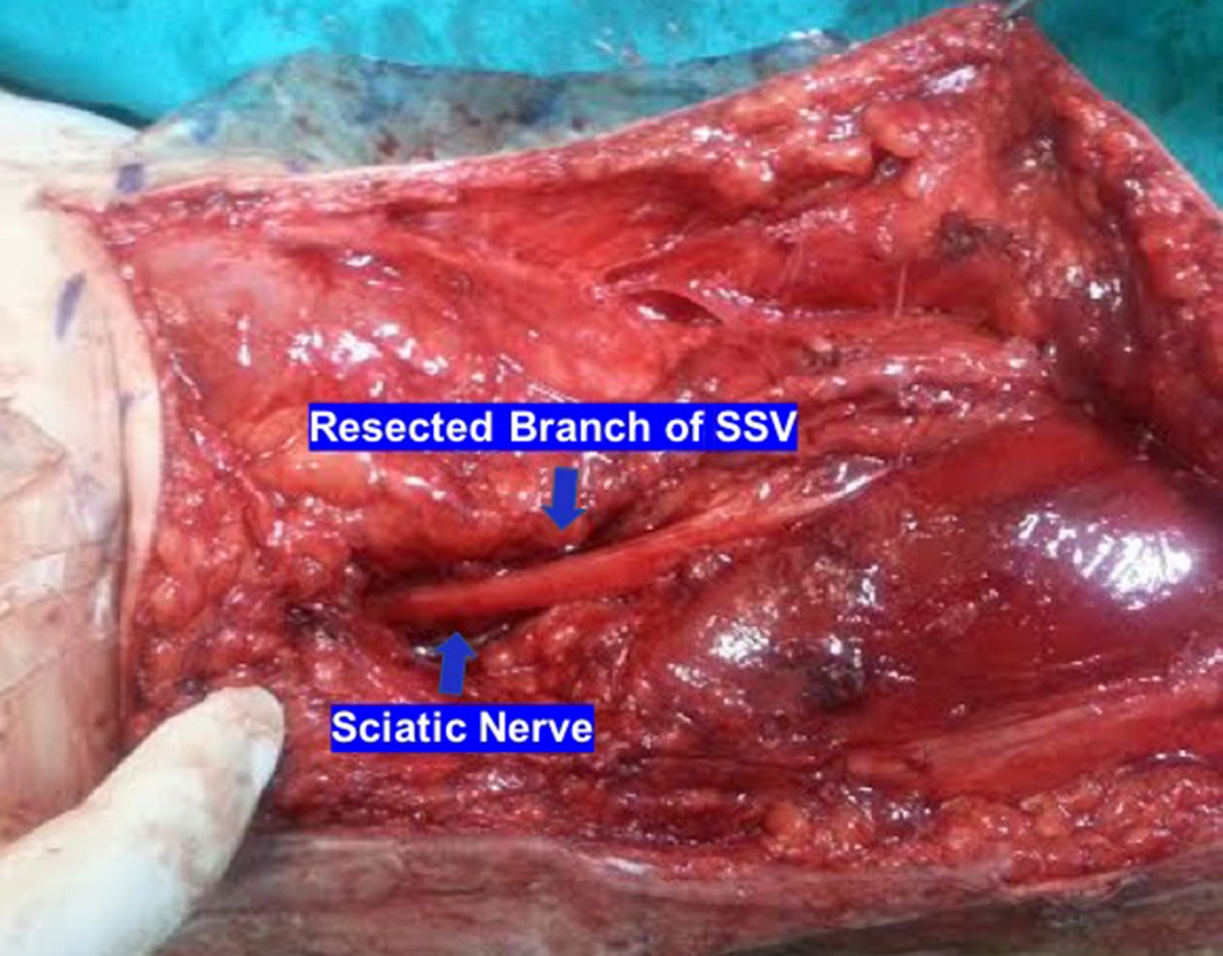
Ligation and resection of the terminal section of the small saphenous vein (SSV) relieved the pressure on the sciatic nerve

Under general anaesthesia, a bilateral posterior approach of popliteal fossa using a lazy S-shaped skin incision was utilized. In both legs, the sciatic nerve was found to be entrapped proximally from its division to the tibial and common peroneal nerves from the terminal branch of the SSV into the PV (Figure 1). The SSV had previously divided into its terminal popliteal branch that ran obliquely and it crossed above the sciatic nerve and Giacomini vein which is a thigh extension of SSV. The terminal section of SSV was subsequently ligated and resected to relieve the pressure on the sciatic nerve (Figure 2). Postoperatively, partial weight-bearing with crutches for two weeks was suggested. After that time, gradual mobilization and return to sports was recommended. At three months from surgery, the patient was pain free and was able to run without facing intermittent claudication. At the latest follow-up one year after surgery, he was able to run long distances as well as sprint and train without any restriction.

## Discussion

Claudication in young patients and athletes is a rare condition that is very difficult to be diagnosed. When the symptoms are induced with exercise, the differential diagnosis includes conditions with muscle-fascia origin (i.e., chronic exertional compartment syndrome, fascial defects, unresolved muscle strain), bone-periosteum origin (i.e., medial tibia stress syndrome, fibular and tibial stress fractures), vascular origin (i.e., vascular claudication, atherosclerotic causes, or popliteal artery entrapment syndrome), and nerve origin (i.e., lumbar disc herniation with referred pain arisen from the low back, nerve entrapment syndromes) [1]. Therefore, an accurate diagnosis requires obtaining a detailed patient history, performing a comprehensive physical examination, and coordinating the appropriate diagnostic exams to further distinguish between the different conditions [4].

The common peroneal, superficial peroneal, and saphenous nerves are the most common nerves at risk for entrapment, while tibial and sciatic nerve compression is quite rare. Abnormal embryologic development leads to several anomalous relations in the popliteal fossa that may apply direct external pressure to the popliteal artery and vein and compress the tibial or sciatic nerves [5]. In our case, vasodilatation during exercise of the termination of the SSV into the PV caused significant local compression of the sciatic nerve and produced bilateral pseudoclaudication in a young athlete. To our knowledge, there has never been a report of bilateral sciatic nerve compression from the terminal section of the SSV.

Multiple variations regarding the termination of SSV have been described. Over one half (60%) of SSVs join the PV within 5 cm of the knee joint. At least one-third, however, enter above the knee joint, and a smaller proportion (5%-10%) join the PVs below the knee joint. Furthermore, and in up to 70% of cases, the SSV may continue in the proximal thigh after communicating with the PV in the popliteal fossa as the Giacomini vein. Although the sciatic nerve is located superficially to sapheno-popliteal junction, we found that it may be “squeezed” if it runs between the termination of SSV and PV [6].

The role of imaging in the diagnosis of nerve entrapment is limited and restricted to exclude other pathologic conditions (e.g., herniated disc) or to identify any vascular or musculoskeletal anatomical abnormalities compromising the nerve function. Popliteal artery entrapment syndrome is excluded by duplex ultrasound analysis of the popliteal artery flow patterns in various foot positions as well as normal angiographic findings. Plain radiographs or computed tomography (CT) scan can rule out any tibial or fibular stress fractures, while MRI imaging is the technique of choice for detecting any soft tissue tumors and lesions. In most cases of nerve entrapment, the EMG and MRI findings are normal and diagnosis is difficult to establish [7]. Furthermore, as the symptoms appear during exercise, treadmill testing should be done in order to clarify the origin, nature, and severity of the claudication.

Surgical exploration of the sciatic nerve should be considered when clinical suspicion of sciatic nerve entrapment is evident and no improvement of symptoms is apparent after four to six months of conservative treatment with nonsteroidal anti- inflammatory drugs (NSAIDs), physiotherapy, rest, and muscular stretching. The nerve is carefully dissected and any potential areas of compression from fibrous bands or veins must be recognized [3]. In our case, the reason for bilateral sciatic nerve pressure was the unusual oblique course of the terminal junction of SSV before it drained into the PV and the position of sciatic nerve between the two veins. Ligation of the terminal branch of SSV and subsequent nerve release led to symptoms resolution and the patient fully returned to sports activities without complaining of any pain or numbness of the lower legs.

## Conclusions

In conclusion, a physician should be aware of rare conditions that may lead to pseudoclaudication in young individuals and athletes. Particularly, the appearance of symptoms during exercise along with normal radiologic and EMG must raise the suspicion of sciatic nerve compression from fibrous bands or abnormal anastomotic veins that may also exist bilaterally. Surgical exploration and nerve release remain the only treatment solution when conservative treatment fails to alleviate the symptoms. In this scenario and after intraoperative confirmation of nerve compression, an excellent postoperative outcome, as well as complete resolution of symptoms, should be expected.
